# Experimental Study of Fiber Pull-Outs in a Polymer Mortar Matrix

**DOI:** 10.3390/ma16093594

**Published:** 2023-05-08

**Authors:** Lihua Wang, Tongshuai Li, Qinghua Shu, Shifu Sun, Chunfeng Li, Chunquan Dai

**Affiliations:** 1 College of Civil Engineering and Architecture, Shandong University of Science and Technology, Qingdao 266590, China; wlh@sdust.edu.cn (L.W.); litongshuai1@163.com (T.L.); sqh_fendou@163.com (Q.S.); 17861698218@163.com (S.S.); 19862206909@163.com (C.L.); 2 Shandong Key Laboratory of Civil Engineering Disaster Prevent and Mitigation, Qingdao 266590, China

**Keywords:** cement mortar, vinyl acetate–ethylene polymer, glass fiber, polypropylene fiber, pull-out load–slip curve, microstructure

## Abstract

In order to study the influence of vinyl acetate–ethylene copolymerization emulsions on the bonding performance of fiber and mortar, mortar materials with different polymer contents were prepared. The optimal mix ratio of the matrix was obtained using a pull-out test with a 0° inclination angle. On this basis, polypropylene fibers and alkali-resistant glass fibers were set at different burial depths (6 mm, 12 mm, and 18 mm) and different burial angles (0°, 30°, 45°, and 60°). The load–displacement curves of two types of fibers pulled out from the polymer mortar were obtained. The test results show that polymer contents of 3% and 5% increase the peak pull-out loads of glass fibers and polypropylene fibers by 16.28% and 30.72% and 7.41% and 27.11%, respectively. When the polymer content is 7%, the peak pull-out load decreases by 1.31% and 24.26%, especially for polypropylene fiber, which significantly weakens the bonding performance between the matrix and the fiber. The pull-out load of glass fibers and polypropylene fibers increases with the increase in the buried depth, and both show tensile failure at 18 mm. As the embedding angle increases, the pull-out load of polypropylene fibers decreases continuously, while the glass fiber shows a higher pull-out load at 30°.

## 1. Introduction

The bonding performance between fibers and a cement matrix is of great significance for understanding the macroscopic mechanical properties and failure modes of fiber-reinforced cement-based materials. Currently, experimental research on the bonding performance of fiber- and cement-based materials is divided into a single-fiber pull-out test, a single-fiber press-out test, and a fiber fracture test [1,2]. The pull-out test considers the bridging effect in the practical application of fibers, and has become the main test method. The research and applications of fiber-reinforced cement-based materials mainly include the following three categories: different fibers and ordinary cement-based materials; modified fibers; and cement-based materials. Fiber and modified cement-based materials have achieved fruitful results.

Ma Yiping [3] carried out the pull-out test on a polypropylene fiber self-cementing matrix, in which the fiber has seven different embedded lengths in the matrix. The test results showed that the interfacial bonding performance between the fiber and the cement matrix is related to the depth of the fiber, and the bonding strength decreases with the increase in the depth of the fiber. Zhang Xianmin et al. [4] compared the pull-out mechanical behaviors of steel fibers, polyvinyl alcohol fibers, polypropylene crude fibers, and impregnated basalt fibers with respect to different factors such as the water–binder ratio and different embedding depths. The experiment found that different fiber types significantly alter the interface bonding behavior. In a comparison of four existing fibers, polypropylene crude fiber exhibited better bonding performances. Xu Lihua et al. [5] used the nanoindentation test to study the mechanical properties of the interfacial transition zone of steel-fiber-reinforced cement-based materials with respect to the water–cement ratio. With the help of microscopic tests, the phase formation of the fiber–cement interface transition zone under different water–cement ratios was obtained. In the hydration products of the interfacial transition zone, with the increase in the water–cement ratio, low-density hydrated calcium silicate with poor mechanical properties is continuously generated, thereby reducing the mechanical properties of the interface. Shannag et al. [6] studied the effects of the matrix compactness, fiber burial depth, and fiber volume fraction on the fibers’ pull-out performance via a single-fiber pull-out test. The results showed that a dense matrix can significantly improve the interface performance, and increases in the fiber burial depth and fiber volume fraction in the matrix can increase the peak pull-out force and pull-out energy. Jeong-Il Choi et al. [7] studied the interfacial bonding strength of basalt fiber and polypropylene with cement-based materials by means of a single-fiber pull-out test and prepared a cement matrix with slag, silica fume, and sand as the mixture. Basalt fibers and polyvinyl alcohol fibers with a depth of about 1 mm were embedded in the matrix for the single-fiber pull-out test. The test results showed that the chemical bonding performance of the interface between the basalt fiber and the cement matrix was strong, and the bonding strength and slip hardening coefficient of the interface between the two fibers and the mixture matrix were determined (the characteristic value of the load–displacement curve of the fiber pull-out test). Jin-Keun Kim et al. [8] studied the effect of fine blast furnace slag content on the interfacial properties of a polyvinyl alcohol single fiber when pulled out in the cement matrix; they proposed a mixing ratio range for high-toughness cement-based materials. Silva et al. [9] studied the effects of curing times, the fiber depth, and the fiber shape on interfacial bonding properties in a single-fiber pull-out test. The results showed that the fiber’s shape played a more important role in promoting interfacial properties. In order to study the possibility of using waste fishing nets as short-fiber-reinforced cement-based materials, Jun Kil Park et al. [10] carried out a single-fiber pull-out test of waste fishing nets. The test results show that the unique bundle structure of the bundled waste fishing nets improves the mechanical anchorage between the fiber and the cement matrix, thereby improving the bonding performance of the fiber, making waste fishing nets a possible reinforcing material. Ali Dalalbashi et al. [11] used three different test devices to carry out a test on steel fiber pull-out mortar in order to explore the influence of different devices on the bond slip of steel fibers. The test results show that the pull–push test setup (pull–push II setup) more accurately reflects the bond slip of steel fibers. During the test, epoxy resin was used to wrap the pull-out end of the steel fibers, which not only prevents the premature failure of the free end of the fiber but also facilitates the connection with LVDT, so as to accurately measure the slip during the pull-out process of the fibers.

P. Di Maida et al. [12] treated polypropylene fibers with nano-silica and then prepared cement-based materials. The interfacial properties between the fiber and the matrix were analyzed by a drawing test and scanning electron microscopy. The results showed that the maximum load and drawn energy of treated polypropylene fibers were higher than those of untreated polypropylene fibers; this is mainly because the hydration products and activity of nano-silica improved the interfacial properties, and the hydration products made the interface transition zone denser. Pi Zhenyu et al. [13] studied the effect of fiber and matrix modification on the microstructure of the interface by modifying the fiber and matrix in turn. It was found that the fiber modification was more favorable for the peak load and average bonding strength, while the matrix modification improved the post-peak load and equivalent bonding strength. When the silica fume content was 10%, the effect of the SiO_2_ content on the bonding performance was relatively small; however, when the silica fume content increased to 20%, the bonding performance of the sample with higher SiO_2_ content was significantly better. Yineth Garcia-Diaz et al. [14] used three surface treatment methods to treat five types of fibers in order to study the improvement effect of fiber modification on the bonding performance of the cement matrix. The experimental results show that, after fiber modification, the microstructure of the mortar matrix improved, the interfacial transition zone between the fiber and the matrix was optimized, and the degree of combinations between them improved. In general, the modification of Fiber 1, Fiber 2, and Fiber 5 by NaOH impregnation is unfavorable for the bonding performance of the fiber. The three modification methods have no significant effects on the bond strength of metal fibers; Fiber 3 showed different modification effects from the other fibers. The three modification methods all improved the bonding performance of the fiber to varying degrees. For the fiber with a depth of 1 cm, the modification effect of NaOH was the best. For the fiber with a depth of 2 cm, the nano-silica treatment method has the best enhancement effect. D. V. Soulioti et al. [15] used zinc phosphate to modify steel fibers to enhance the bonding performance between the steel fiber and the cement matrix. The test results show that, after the modification of straight steel fibers, the ZnPh crystal that formed on the surface of the fiber increased the contact area between the matrix and the fiber, which increased the pull-out load and bond strength of the fiber. Jon-Pil Won et al. [16] used hydrophilic maleic-anhydride-grafted polypropylene to impregnate PET fibers and carried out hydrophilic treatments to improve the bonding performance between the PET fiber and cement-based materials. The figure-of-eight-shaped tensile specimen was used for the bonding performance test. The results showed that mPP helps to improve the bonding between the PET fiber and the mortar, and the bonding strength is the best when the concentration of mPP is 15%. When the concentration increased to 20%, the surface layer of the PET fiber cracked due to the high concentrations, which weakened the performance of the fiber itself. During the pull-out test, the fiber was prematurely broken.

M. G. Alberti et al. [17] carried out a pull-out test of polyolefin fibers from a cement base and recorded the load–displacement curve of the test. The performance of the interface between the fiber and the cement base was studied by using different pull-out angles. The test results show that the maximum peak load occurs when the pull-out angle is 45°. When the pull-out angle is less than 45°, the test phenomenon comprised mortar fracture peeling. Qi et al. [18] studied the mechanical properties of a series of inclined straight steel fibers and end-hook steel fibers pulled out from a UHPC matrix and proposed using the energy dissipation index and bond strength index to evaluate the bonding properties of the fiber matrix. Lee Yun et al. [19] studied the effect of steel fiber embedding angles on the tensile properties of ultra-high-strength cementitious composites. The results show that the peak load appears at inclination angles of 30° and 45° when the steel fiber is pulled out, and the peak slip increases as the fiber is oriented at a more inclined angle. A pull-out behavior model considering fiber inclination was proposed. Xinxin Ding et al. [20] also studied the bonding properties of steel fibers and mortars and discussed the influence of the fiber inclination angle and fiber spacing on the bonding strength. The results show that, when the fiber inclination angle is greater than 30°, the pull-out process will cause some mortars at the cross section of the mortar to peel off at a certain depth, thus reducing the bonding properties of steel fibers and mortars. At the same time, when fiber spacing is small, the mortar near the interfacial transition zone will be scraped during the fiber pull-out process, affecting the bonding performance of the adjacent fiber. Arturs Lukasenoks et al. [21] conducted a single-fiber pull-out test using carbon fibers with different buried depths and inclination angles to study the bonding performance between the fiber and the matrix. The test results showed that the fiber inclination angles of 15° and 30° are better than the inclination angle of 0°. When the fiber inclination angle increases to 45°, the bond strength of the fiber depends on the performance of the cement matrix.

By modifying the fiber, the bonding performance between the fiber and the matrix is improved, which only affects the performance of the smaller area near the interface transition zone and cannot compensate for the inherent defects of ordinary mortar materials. With the development of polymer materials science and researchers’ in-depth understanding of the relationship between the material’s structure and properties, adding polymer materials such as polymers to cement mortars to prepare a modified mortar has become a focus of research in engineering, with the aim of enhancing the toughness and bonding properties of cement mortar. Based on the complementary effects of organic polymers and cement-based materials, interpenetrating three-dimensional grid structures are formed inside cement-based materials to improve the performance of these materials [22].

Jianhui Liu et al. [23] improved the performance of the interfacial transition zone between steel fibers and a concrete matrix by using super-absorbent resin. The experimental results show that the addition of SAP increases the density of the matrix near the steel fiber, increases the extrusion force between the fiber and the matrix, and thus increases the pull-out load of the fiber. Among the studied materials, a low content level of SAP with a fine particle size shows the most obvious improvements in peak load and bond strength. This is mainly due to the water release effect of SAP in the later stage, which leaves holes in the interface’s transition zone. The larger the SAP particle size and the higher the content, the more harmful pores will be produced in the later stage, which will seriously weaken the compactness of the transition zone. Chan-Gi Park et al. [24] used styrene–butadiene latex to modify mortar in order to study the bonding properties of modified mortars and polypropylene crude fibers. The test results show that styrene–butadiene latex helps fill the pores inside the mortar. At the same time, the unique film-forming characteristics of the polymer wrap fine aggregates inside the mortar, improve the overall compactness of the matrix, and significantly improve the bonding between the fiber and the matrix. When the content of styrene–butadiene latex exceeds 20%, the hydration reaction of the cement is delayed, and the bond strength is weakened. Shaikh F U A [25] prepared two different types of geopolymers and used the pull-out test to study the pull-out behaviors of steel fibers with different degrees of bending at the ends. The test results showed that the steel fiber and heat-cured fly ash geopolymer (HGP) showed a higher pull-out load, while the steel fiber and ambient air-cured fly ash geopolymer (AGP) showed stronger toughness. Based on the above research results, the modification of cement-based materials and the enhancement of the bonding performance between fibers and cement-based materials are more effective in improving the overall performance of cement-based composites. Considering that there are few experimental studies on the bonding performance between fibers and polymer-modified mortars, in this paper, the interfacial bonding properties between fibers and a matrix were studied using a fiber pull-out test and by considering three factors: the polymer content, the fiber embedding depth, and the inclination angle of the fiber. This study will provide a basis for preparing cement-based composites with good performance.

## 2. Materials and Methods

### 2.1. Specimen Preparation

The P.O42.5 cement used in this test meets the requirements of Chinese standard GB 175-2020 [26]; ISO cement standard sand was used as a fine aggregate, and the mud content of the sand was less than 0.2%. The test used VAE emulsions produced by a company in Beijing, and the relevant performance parameters are shown in Table 1. The polyether concrete-defoaming agent DE-1226, which is a milky white liquid with a solid content of 65%, was used. Anti-Crack HD alkali-resistant glass fibers and polypropylene fibers were used. The physical properties of the fibers are shown in Table 2.

The VAE emulsion was used to modify the cement mortar. The mortar matrix used in the test is shown in Table 3. The cementitious material is the sum of the non-volatile substances in cement and the VAE emulsion. The total water consumption is the sum of the added water and the water content in the VAE emulsion. The water–binder ratio is 0.45, and the cement–sand ratio is 1:2.5. The VAE emulsion content is 0, 3%, 5%, and 7% of the cement content. In addition, the VAE emulsion produces many bubbles in the mortar matrix due to the active components on the surface during the mortar mixing process, which affects the strength of the mortar. Therefore, the polyether concrete-defoamer DE-1226 was used to reduce the number of bubbles in the mixing process; the amount of defoamer is 3.5% of the amount of cement.

### 2.2. Fiber Pullout Test

**Figure 1:**
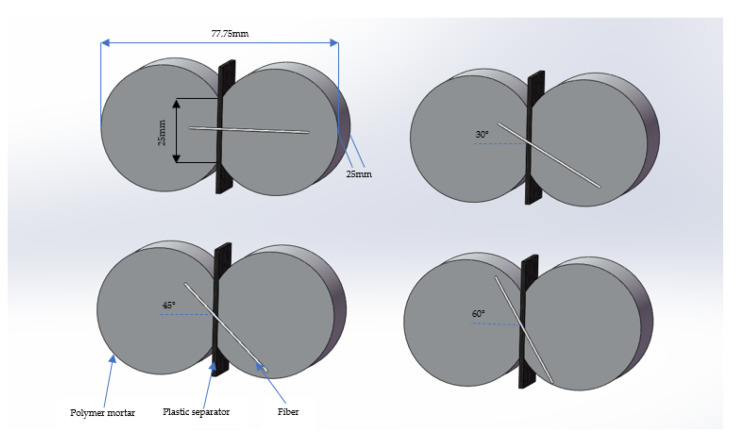
Fiber pull-out specimen schematic diagram.

The fiber pull-out test is a common method for testing the properties of the fiber and the matrix. In this paper, all fiber pull-out specimens were prepared by an 8-shaped test mold. To ensure the reliability of the data, 6 specimens were prepared in each group, and the results were averaged for data processing. The fiber was placed on a pre-prepared plastic partition so that the embedded end length and fiber inclination angle could be accurately measured with a measuring instrument. First, test water was added to the mortar mixer, and then cement was added and stirred at low speeds for 30 s; the sand was added and stirred at high speeds for 60 s; finally, the VAE emulsion and defoamer were added and stirred at low speeds for 60 s. The mixed polymer mortar was poured into the test mold and placed in a curing box for 36 h; then, the mold was removed, and standard curing was carried out in the curing box. Figure 1 is a schematic diagram of the preparation of the test pieces used in this paper.

The test was carried out according to Chinese standard CECS13: 2009 [27], and a 30 kN hydraulic servo testing machine was used for testing. First, the fixture of the testing machine was fixed, and the tensile test piece was placed between the two fixtures to ensure that the test piece was centered and in a micro-stress state. The testing machine was started, and then the test started after the data of the testing machine were cleared. According to the loading scheme, the displacement loading mode was adopted, and displacement loading was carried out at a rate of 0.5 mm/min. The test machine automatically records the load–displacement curve during the test. According to the relevant research [28], the interfacial bond strength between the fiber and mortar is calculated according to Formula (1):

(1) $$\tau =\frac{F}{\pi \cdot d_{f}\cdot l_{em}}$$

where τ is the average bond strength, F is the fiber pull-out load, $d_{f}$ is the fiber diameter, and $l_{em}$ is the fiber embedding length.

## 3. Results and Discussion

### 3.1. The Effect of VAE Emulsion Content on Bonding Properties

**Figure 2:**
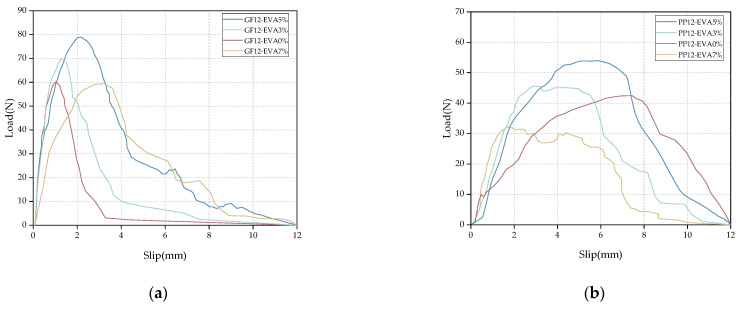
Load–displacement curves under different VAE contents: (**a**) glass fiber and (**b**) polypropylene fiber.

**Figure 3:**
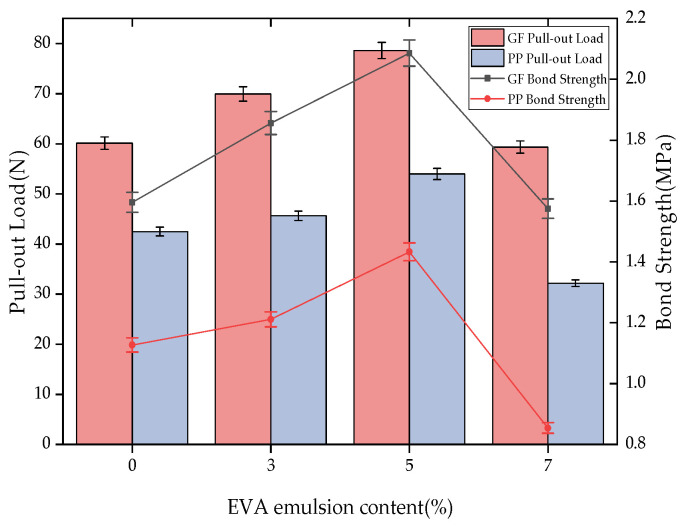
The relationship between the VAE emulsion content and the average bond strength.

Figure 2 shows the pull-out curves of the glass fibers and polypropylene fibers with different VAE emulsion contents. It can be seen from the figure that the effect of VAE emulsion contents on the pull-out curves is basically the same for the two fibers. With the increase in VAE emulsion content, the peak pull-out load increases first and then decreases. When the VAE emulsion content is 5%, the peak pull-out load of the two fibers reaches the maximum value. Table 4 summarizes the pull-out test results for the glass fibers and polypropylene fibers under different matrix mix ratios. In order to compare the average bond strength and pull-out load of the two fibers with the change in VAE content, the test results are shown in Figure 3. From the test results, it is observed that, under the same buried depth and inclination angle, the peak pull-out load of the glass fibers increased by 16.28%, 30.72%, and −1.31%, and the peak pull-out load of the polypropylene fibers increased by 7.4%, 27.11% and −24.26%, with one group as the control group. The 7% VAE emulsion content has a negative growth effect on the pull-out load of both fibers. The average bond strength and pull-out load of the glass fibers are greater than those of polypropylene fibers in the entire range, indicating that alkali-resistant glass fibers and polymer mortars exhibit excellent bonding properties. Therefore, the bonding performance between the fiber and polymer mortar mainly depends on the performance of the matrix itself. An appropriate amount of VAE emulsion can improve the performance of the interfacial transition zone in the mortar matrix, and the hydration degree of the polymer mortar embedded near the fiber is better, so the mortar can wrap the fiber better, and the bonding performance is improved. However, the unique film-forming effect of the VAE emulsion limits the hydration reaction process of cement to a certain degree. Therefore, when the VAE emulsion content is 7%, the strength increase rate of the matrix begins to slow down, resulting in a decrease in the peak pull-out load and average bond strength.

In summary, the bonding properties of the matrix prepared by a ratio of 3 with glass and polypropylene fibers are superior. Therefore, on the basis of the ratio of 3, pull-out tests of the glass and polypropylene fibers with different embedding depths and inclination angles were carried out.

### 3.2. The Effect of Fiber Angle and Depth on the Bonding Properties

#### 3.2.1. Analysis of the Failure Pattern

**Figure 4:**
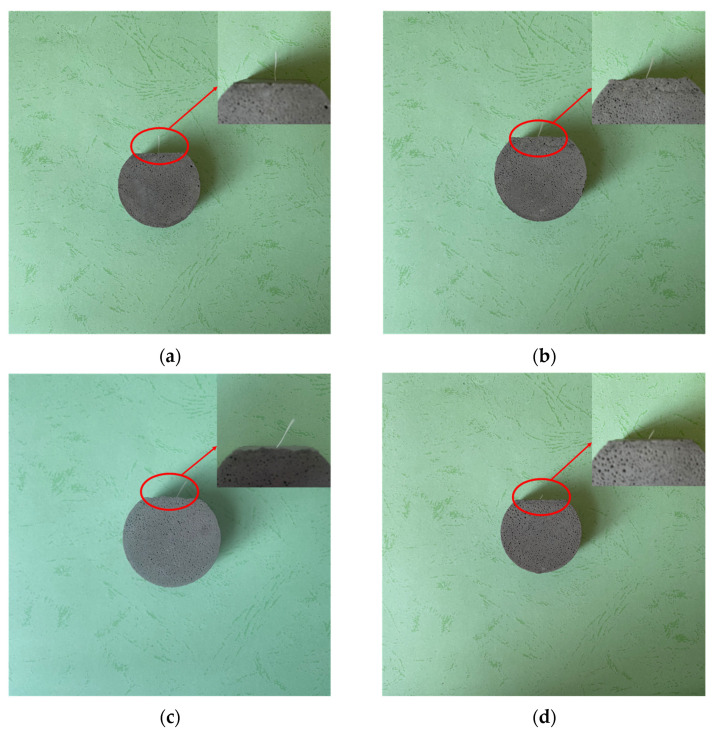
Failure mode of single-fiber multi-angle pull-out specimens: (**a**) 0°, (**b**) 30°, (**c**) 45°, and (**d**) 60°.

The failure mode of the specimen is shown in Figure 4, and the failure mode of the fiber multi-angle pull-out specimen is divided into two types. The first is the failure mode of the completely pulled-out fiber; the second is the failure mode of fiber breakage. The first form of failure fully reflects the bonding performance between the fibers and the matrix. When the bonding performance between the fiber and matrix is too high, the second failure mode is generated.

The reason for this phenomenon is that the chemical bonding force between the fiber and the mortar matrix caused by the high strength of the matrix itself is large, and the pull-out load exceeds the fiber. In addition, the embedded depth of the fiber is an essential reason for this failure mode. Due to the increase in the embedded depth of the fiber, the friction between the interface and the fiber during the pull-out process makes the effective area of the fiber decrease, and the maximum withstanding tensile force continues to decrease so that the fiber is not completely pulled out of the mortar matrix. At the same time, with different inclination angles of the fibers in the matrix, the failure modes are also different. When the inclination angle increases, the friction-weakening effect of the interface on the fiber surface is more pronounced during the fiber pull-out process. When the specific failure mode is about 45° to 60°, the fiber causes the matrix around the pull-out end to peel off during the pull-out process. The larger the inclination angle, the more obvious the peeling phenomenon.

#### 3.2.2. Pull-Out Results

**Figure 5:**
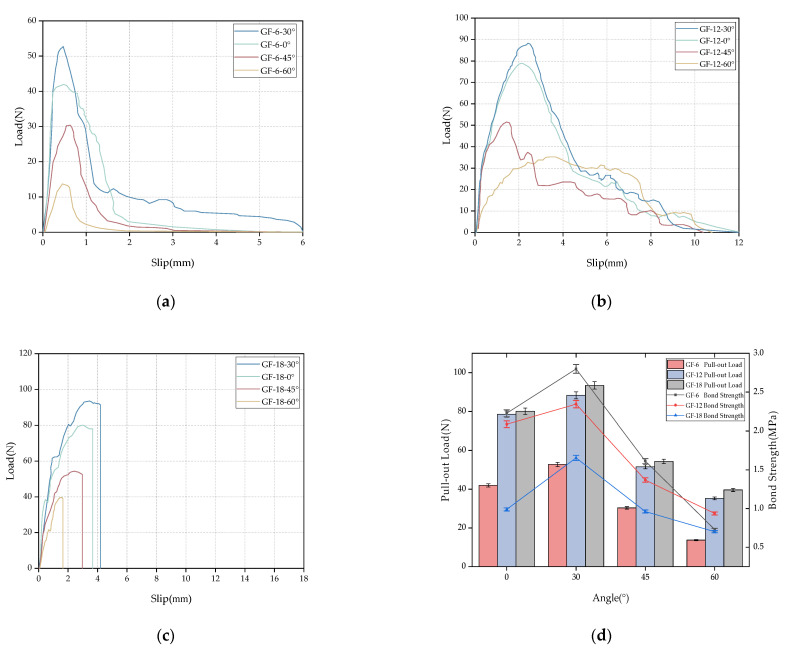
Relationship between bonding properties and the glass fiber depth and angle: (**a**) 6 mm load–displacement curve, (**b**) 12 mm load–displacement curve, (**c**) 18 mm load–displacement curve, and (**d**) variation trend of the drawing load and average bonding strength.

Figure 5 shows the load–displacement curve of the glass fiber specimen under multiple angles and multiple buried depths. When the fiber embedding length is 6 mm and 12 mm, the failure mode of the specimen is that whereby the fiber is completely pulled out. It can be observed from the curve that the change in the fiber angle has a significant effect on the pull-out load. Table 5 shows the pull-out load and bond strength of glass fiber and polypropylene fiber at different embedding depths and inclinations. Taking the fiber with an embedding length of 12 mm as an example, the peak pull-out load increases by 12.40% and 71.36%, respectively, at the embedding angle of 30° compared with 0° and 45°. This is mainly due to the increase in the angle, which enhances the friction between the fiber and the matrix. At the same time, the pull-out channel of the inclined angle produces a resistance effect on the fiber at the outlet, so it produces a larger pull-out load than the 0° fiber. At the same time, it is observed that, for the glass fiber specimens, the effect of increasing the inclination angle on the pull-out load gradually decreases with the further increase in the fiber embedding angle. This is mainly because of the resistance generated by the matrix at the pull-out end. The damage to the fiber surface will further increase with the increase in the embedding depth, causing the matrix at this part to peel off during the fiber pull-out process, thereby reducing the actual embedding length of the fiber and the corresponding increase in the pull-out load. With the increase in the fibers’ embedding length, it is increasingly difficult for the fibers to be completely pulled out from the matrix. The pull-out specimens under 18 mm are dominated by fiber pull-outs, which is reflected in the curve when the load suddenly decreases to 0 under a certain displacement. During the test, it was also found that the resistance of the matrix at the pull-out end produced different performances at the low buried depth of 18 mm. At an angle of 60°, the matrix did not peel off, and the fiber was first pulled off.

Figure 5a–c shows a summary of the glass fiber pull-out test results for different buried depths and inclination angles. In order to compare the changing trends of the average bond strength and pull-out load of the two fibers for different embedding depths and inclination angles, the test results are shown in Figure 5d. It can be observed from the figure that the increase in the fiber embedding depth helps increase the peak pull-out load. Under the fiber inclination angle of 0°, the peak pull-out loads at 12 mm and 18 mm burial depths are 87.34% and 90.98% higher than the peak pull-out load at a 6 mm burial depth, respectively. This indicates that the toughening effect of long fibers on the matrix is better than that of short fibers, but the bond strength corresponding to the load decreases with the increase in the fiber embedding depth. When the inclination angle is 0°, the bond strength decreases by 6.3% and 55.6% with the increase in buried depth. This phenomenon is mainly due to the relatively uniform stress distribution in the fiber depth direction under the shallow buried depth of the fiber. However, with the increase in the buried depth, the stress at the fiber pull-out end and the buried end is relatively large, and the stress at other positions in the longitudinal direction is small; moreover, the stress distribution is highly uneven. With the increase in the fiber inclination angle, the phenomenon of uneven stress distribution caused by the initial deeper buried depth improves, and the difference in bond strength under the three buried depths of the fiber is gradually reduced.

**Figure 6:**
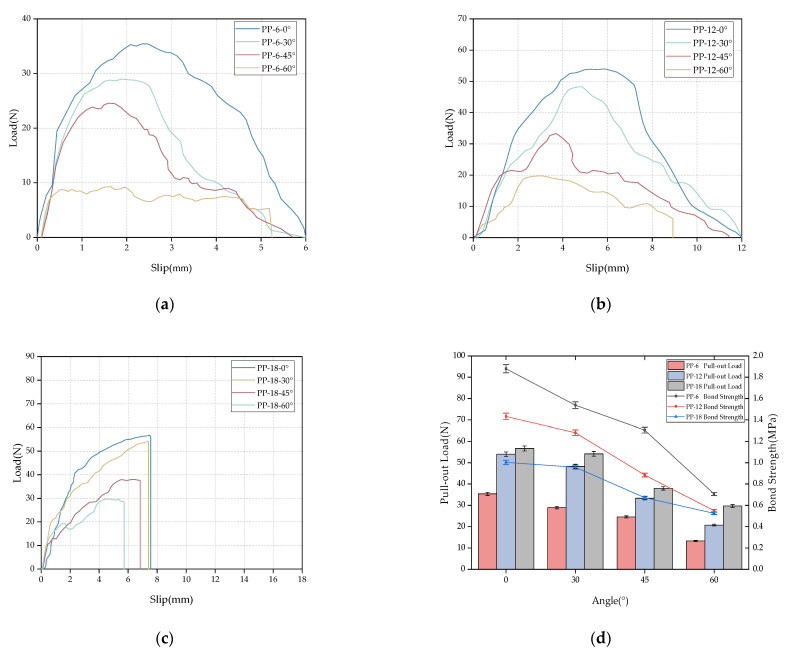
The relationship between the bonding properties and the depth and angle of the polypropylene fiber: (**a**) 6 mm load–displacement curve, (**b**) 12 mm load–displacement curve, (**c**) 18 mm load–displacement curve, and (**d**) the changing trend of the pull-out load and average bond strength.

Figure 6 shows the multi-angle and multi-depth pull-out curve of the polypropylene fibers. From Figure 6b, it is observed that, due to the large chemical bonding force between the mortar matrix, the pull-out load exceeds the tensile strength of the polypropylene fiber, resulting in the fiber pull-out failure of the 12 mm polypropylene fiber at 60°. At the same time, compared with the load–displacement curve of the glass fiber, it can also be found that the increase in the angle has certain weakening effects on the pull-out load of the polypropylene fiber. The increase in the peak load caused by the resistance of the matrix at a low angle is not reflected for the polypropylene fiber specimen. On the contrary, the strength of the fiber itself is weakened due to the scraping of the fiber’s surface by the matrix at the outlet during the pull-out process. Figure 6c shows the load–displacement curve of an 18 mm polypropylene fiber. Compared with the glass fiber, the load–displacement curve of the polypropylene fiber with 45° and 60° inclination angles fluctuates briefly near the peak, which indicates that the mortar at the outlet is crushed or peeled off.

From Figure 6d, it is observed that the pull-out load of the polypropylene fiber decreases gradually with the increase in the fiber inclination angle at the three buried depths. At the same time, with the increase in the fiber burial depth, the pull-out load at the inclination angles of 0° and 30° increased; however, with the further increase in the fiber inclination angle, the pull-out load of the polypropylene fiber at a burial depth of 18 mm began to decrease significantly. This is mainly due to the matrix’s peeling at the pull-out end during the fiber pull-out process at a large inclination angle. In addition, with the increase in the fiber embedding depth, the average bond strength showed a downward trend, which also occurred in the polypropylene fibers. When the inclination angle is 0°, the bond strength decreases by 23.78% and 46.65% with the increase in the buried depth.

**Figure 7:**
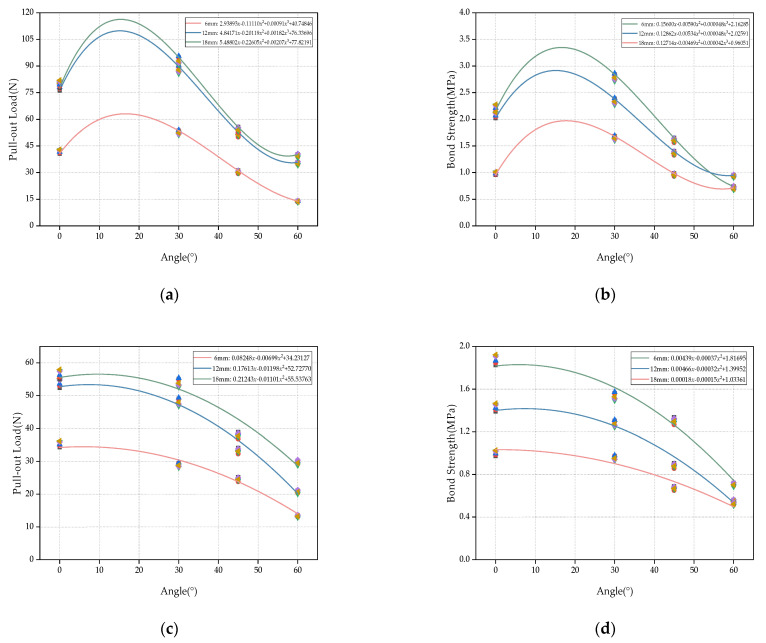
Fitting curves of fiber pull-out load and bond strength with inclination angle: (**a**) glass fiber load fitting curve, (**b**) glass fiber strength fitting curve, (**c**) polypropylene fiber load fitting curve, and (**d**) polypropylene fiber strength fitting curve.

Figure 7 is based on the least square method; considering the different embedding depths of the fiber, the fitting curve of the glass fiber pull-out load and bond strength with the fiber embedding angle is obtained. From Figure 7a,b, it can be seen that the curve obtained by cubic polynomial fitting is more accurate. The three fitting curves shown in Figure 7a represent the relationship between the pull-out load and the inclination angle under different fiber embedding depths. The fitting results of Figure 7a are found. The fitting curves of 18 mm and 12 mm are close to each other, indicating that, after 12 mm, the influence of the fiber embedding depth on the pull-out load of glass fiber debonding tends to be stable. Figure 7c,d is the fitting curve of pull-out load and bond strength of polypropylene fibers with the fiber inclination angle. At a fiber inclination angle of about 60°, the change trend of bond strength becomes gentle. Figure 7c,d is the fitting curve of the pull-out load and bond strength of the polypropylene fiber with the fiber embedding angle considering the different embedding depths of the fiber. The fitting curve R^2^ of the three groups of pull-out loads is above 94%, and the fitting curve R^2^ of the bond strength is above 95.5%. The model can fit the data well, which is helpful for the comparison and verification of the subsequent experimental results.

## 4. Conclusions

By conducting a single-fiber pull-out test, the bonding properties of the fiber and polymer mortar under different contents of VAE emulsions were studied. The pull-out tests of different embedding depths and embedding angles of glass and polypropylene fibers were carried out using an optimal content of VAE, and the following conclusions were obtained.

(1) With the increase in the VAE emulsion content, the tensile load and average bond strength of the glass fiber and the polypropylene fiber increased continuously. Compared with the control group without VAE emulsions, the pull-out loads of 3% and 5% increased by 16.28% and 30.27% and 7.41% and 27.11%, and the bond strength increased by 16.29% and 30.70% and 7.45% and 27.15%, respectively. When the VAE content was 7%, the cement hydration reaction slowed down to a certain extent, which is not conducive to improving the bond strength. The pull-out load is reduced by 1.31% and 24.26%, and the bond strength is reduced by 1.32% and 24.22%.

(2) During a single-fiber pull-out test, different fiber buried depths and inclination angles show different failure modes. For glass fibers, with the increase in the fiber buried depth, the pull-out load increased continuously. At a buried depth of 18 mm, the fiber presented the failure form of the pull-out test. Under the same buried depth, when the inclination angle of the fiber was below 30°, it played a positive role in the peak load of the fiber pull-out. With the increase in the angle, when the inclination angle is above 45°, the excessive inclination angle causes the end matrix to peel off during the pull-out, which reduces the effective buried depth of the fiber and reduces the pull-out load.

(3) Both fibers show enhanced peak stress when the inclination angle is small. Under different buried depths and inclination angles, the damage caused to the polypropylene fiber is slightly different from that seen in the glass fibers. This is mainly due to the low strength and elongation of polypropylene fibers, resulting in the pull-out failure of the fibers at a buried depth of 6 mm and an inclination angle of 60%.

## Figures and Tables

**Table 1:** VAE emulsion performance index.

pH	Nonvolatile Matter (%)	Viscosity (mPa.s)	Dilution Stability (%)	Ethylene Content (%)
6.5	54.5	500~1000	≤3.5	16 ± 2

**Table 2:** Mechanical properties of the fibers.

Type of Fiber	Length (mm)	Density (kg/m^3^)	Diameter (mm)	Tensile Strength (MPa)	Elastic Modulus (MPa)
GF	6 12 18	2680	0.3	1700	72,000
PP		930	0.3	556.9	6822.3

GF stands for glass fiber; PP stands for polypropylene fiber.

**Table 3:** Mixture proportions.

Code	Cement/g	Water/g	Sand/g	VAE/g	Defoaming Agent/g
Matrix 1	510.00	229.50	1275.00	0	0
Matrix 2	510.00	216.47	1313.25	28.33	0.98
Matrix 3	510.00	207.78	1338.75	47.22	1.65
Matrix 4	510.00	199.09	1364.25	66.11	2.31

**Table 4:** Summary of pullout test results.

Code	Glass Fiber				Polypropylene Fiber			
	P(N)	SD	τ (MPa)	SD	P(N)	SD	τ (MPa)	SD
Matrix 1	60.130	1.231	1.596	0.033	42.475	0.869	1.127	0.023
Matrix 2	69.921	1.431	1.856	0.038	45.620	0.934	1.211	0.025
Matrix 3	78.601	1.609	2.086	0.043	53.989	1.105	1.433	0.029
Matrix 4	59.340	1.215	1.575	0.032	32.170	0.658	0.854	0.017

SD, standard deviation.

**Table 5:** Summary of fiber pull-out test results with different buried depths and inclination angles.

Code	Glass Fiber				Polypropylene Fiber			
	P(N)	SD	τ (MPa)	SD	P(N)	SD	τ (MPa)	SD
T1	41.957	0.725	2.227	0.038	35.419	0.859	1.880	0.046
T2	78.601	1.105	2.086	0.029	53.989	1.609	1.433	0.043
T3	80.130	1.160	0.989	0.021	56.673	1.640	1.003	0.020
T4	52.733	0.593	2.799	0.031	28.976	1.079	1.538	0.057
T5	30.370	0.503	1.612	0.027	24.586	0.622	1.305	0.033
T6	13.716	0.273	0.728	0.014	9.326	0.281	0.495	0.015
T7	88.344	0.988	2.345	0.026	48.269	1.808	1.281	0.048
T8	51.554	0.681	1.368	0.018	33.308	1.055	0.884	0.028
T9	35.269	0.425	0.936	0.011	20.753	0.722	0.551	0.019
T10	93.470	1.109	1.654	0.020	54.169	1.913	0.958	0.034
T11	54.347	0.777	0.962	0.014	37.980	1.112	0.672	0.020
T12	39.615	0.608	0.701	0.011	29.700	0.811	0.525	0.014

SD, standard deviation.

## Data Availability

Data are contained within the article.
